# Ranking of Health Problems and Prioritization of Cancer Education Topics by African American Communities in South Carolina

**DOI:** 10.1007/s13187-024-02543-5

**Published:** 2024-12-21

**Authors:** E. Sylvia Melikam, Gayenell S. Magwood, Marvella Ford, Judith Salley, Latecia Abraham-Hilaire, Joni Nelson, Audrey McCrary-Quarles, Cammie Berry, Delaram Sirizi, Kathleen B. Cartmell

**Affiliations:** 1 Department of Public Health Sciences, Clemson University, 513 Edwards Hall, Clemson, SC 29634, USA; 2 Department of Biobehavioral Health and Nursing Science, College of Nursing, University of South Carolina, 1601 Greene St., Columbia, SC 29208, USA; 3 Cancer Disparities, Department of Public Health Sciences, Hollings Cancer Center, Medical University of South Carolina (MUSC), 86 Jonathan Lucas Street, Charleston, SC 29425, USA; 4 Department of Biological & Physical Sciences, South Carolina State University, 300 College Street, Orangeburg, SC 29117, USA; 5 Medical University of South Carolina, Academic Affairs Faculty, MUSC Library-PICO, 171 Ashley Avenue, Charleston, SC 29425, USA; 6 Division of Population Oral Health, James B. Edwards College of Dental Medicine, Medical University of South Carolina, 173 Ashley Avenue BSB 127, Charleston, SC 29425, USA; 7 Department of Health Sciences & Physical Education, South Carolina State University, 300 College Street, Orangeburg, SC 29117, USA; 8 Office of Institutional Research, South Carolina State University, 300 College Ave, Orangeburg, SC 29117, USA

**Keywords:** Cancer education, Prevention, African Americans, Disparities

## Abstract

Despite landmark breakthroughs in cancer research, African American adults (AA) bear the highest cancer burden compared to other racial groups in the United States (US). AA adults have twice the likelihood of dying from prostate and uterine cancers compared to White adults, suggesting that there are fundamental issues yet to be addressed when developing and implementing cancer-preventative programs for AA communities. Community-based participatory research (CBPR) empowers community members to identify and prioritize their health problems and preferred strategies to tackle these issues. In alignment with the CBPR approach, the South Carolina Cancer Disparities Research Center (SC CADRE) undertook a study to inform cancer research priorities and interventions. A survey designed by the SC CADRE team to assess perceptions about health problems (cancer risk factors), prioritization of cancer education topics, and attitudes related to cancer prevention was completed by predominantly AA community members in South Carolina (*N* = 179). Participants had a mean age of 51.59 ± 16.53 years; the majority were AA (72.49%), females (76.44%), had bachelor’s/graduate degrees (66.29%), and were from the Lowcountry coastal region of the state (85.26%). Obesity emerged as the greatest health concern, followed by poor diet and low physical activity. The top three priorities for cancer education were to learn about causes of cancer, strategies for healthy eating, and how to access healthcare. These findings could inform cancer education and intervention programs to address the top priority health needs identified by AA communities in South Carolina. They may also be relevant in other states, especially in rural southern parts of the USA.

## Introduction

African Americans (AA) have a higher overall cancer death rate compared to the overall US population [1]. Based on 2015–2019 cancer mortality data, the likelihood of prostate cancer-related mortality was twice as high among AA men compared to White men [2]. Similarly, the odds of dying from breast cancer were 40% higher among AA women compared to White women, and the odds of dying from uterine cancer for AA women were twice as high compared to White women [2, 3]. The observed disparities are associated with a relatively higher prevalence of cancer risk factors, poor access to adequate healthcare services, and late diagnosis [4, 5]. Furthermore, delayed diagnosis and treatment have been associated with less favorable outcomes, poor quality of life, and lower survival rates among AA cancer patients [4, 6].

Research studies have reported a lower rate of participation in cancer research among racial minority groups [1]. Some reasons for the low participation of minority groups in cancer prevention programs and research include the inaccessibility to cancer screening or trial sites, medical mistrust, and lack of information [1, 7, 8]. The underrepresentation of groups that suffer these malignancies the most in research undermines the effectiveness of innovative strategies for cancer prevention and leads to the persistence of health disparities [8].

To improve minority inclusion in research and health programs, researchers can partner with key community stakeholders to develop their research concepts from the needs assessment phase, study design, initiation, and implementation through translation stages [9]. Obtaining meaningful input from the community can ensure that new programs are contextually relevant and meet the needs of communities. This community-based participatory research (CBPR) approach can increase the credibility of research for community members. It can also support participant enrollment and retention rates and the usefulness of and value placed by community members on the findings from the research [1, 9]. Thus, CBPR approaches can help to address cancer disparities by circumventing some obstacles to disseminating and translating research findings, considering that meaningful community buy-in and trust can be secured from the onset of the research effort [9].

Consequently, this study had three aims: (1) to identify which cancer risk factors are considered topmost health problems among racial minority communities in South Carolina; (2) to obtain their input regarding prioritization of cancer education topics; and (3) to assess their attitudes about cancer prevention.

## Methods

A descriptive cross-sectional survey was conducted between August 2020 and December 2021 as a partnership between the Community Outreach and Planning and Evaluation Cores of the South Carolina Cancer Disparities Research Center (SC CADRE), sponsored by the National Cancer Institute (NCI) of the National Institutes of Health (NIH) U54-CA210963-01A1 [1, 10]. We previously published a manuscript with data from the same survey project that reported on community preferences for participating in cancer research [10].

### Study Participants

The SC CADRE [10] researchers leveraged already established networks within the various communities in South Carolina (SC) to create awareness about the research and facilitate recruitment of individuals (≥ 18 years) from across the four SC regions (Lowcountry, Midlands, Pee Dee and Upstate) via convenience sampling. This sampling method was used because the study was conducted during the pandemic, which resulted in limited access to resources required for more elaborate sampling techniques. Participants were given information about the nature and objectives of the study, and informed consent was obtained from them before enrollment. The participants were also informed of the confidentiality of the data they provided and their rights to leave the study without penalty.

### Data Collection

A survey was administered to collect data on participants’ demographics, ranking of modifiable cancer risk factors relevant to their community, prioritization of cancer education topics of interest, and attitudes toward cancer prevention. To rank the most important health concerns (risk factors), a Likert scale was used to ask participants to rank health problems from 1 (least problematic) to 5 (most problematic). Similarly, for prioritization of specific cancer education topics in their respective communities, a Likert scale was used, which ranged from 1 (not important) to 5 (very important). Data were analyzed using SAS statistical software version 9.4 [11]. Demographic items were presented as frequencies and percentages and age as mean (standard deviation), while the ranking and prioritization were determined based on averages. The risk factor with the highest average score (out of a maximum of 5) was ranked the most important health concern, while the risk factor with the lowest average score was ranked the least important health problem. The same approach was applied to prioritizing cancer education topics of interest.

## Results

A total of 179 individuals completed the survey. The mean age of participants was 51.59 ± 16.53 years; most participants were females (76.44%), African Americans (72.49%), had a bachelor’s or a graduate degree (66.29%), and resided within the Lowcountry region (mostly Orangeburg and Charleston counties) of South Carolina (85.26%) (Table 1).

### Participants’ Ranking of Health Concerns

**Fig. 1:**
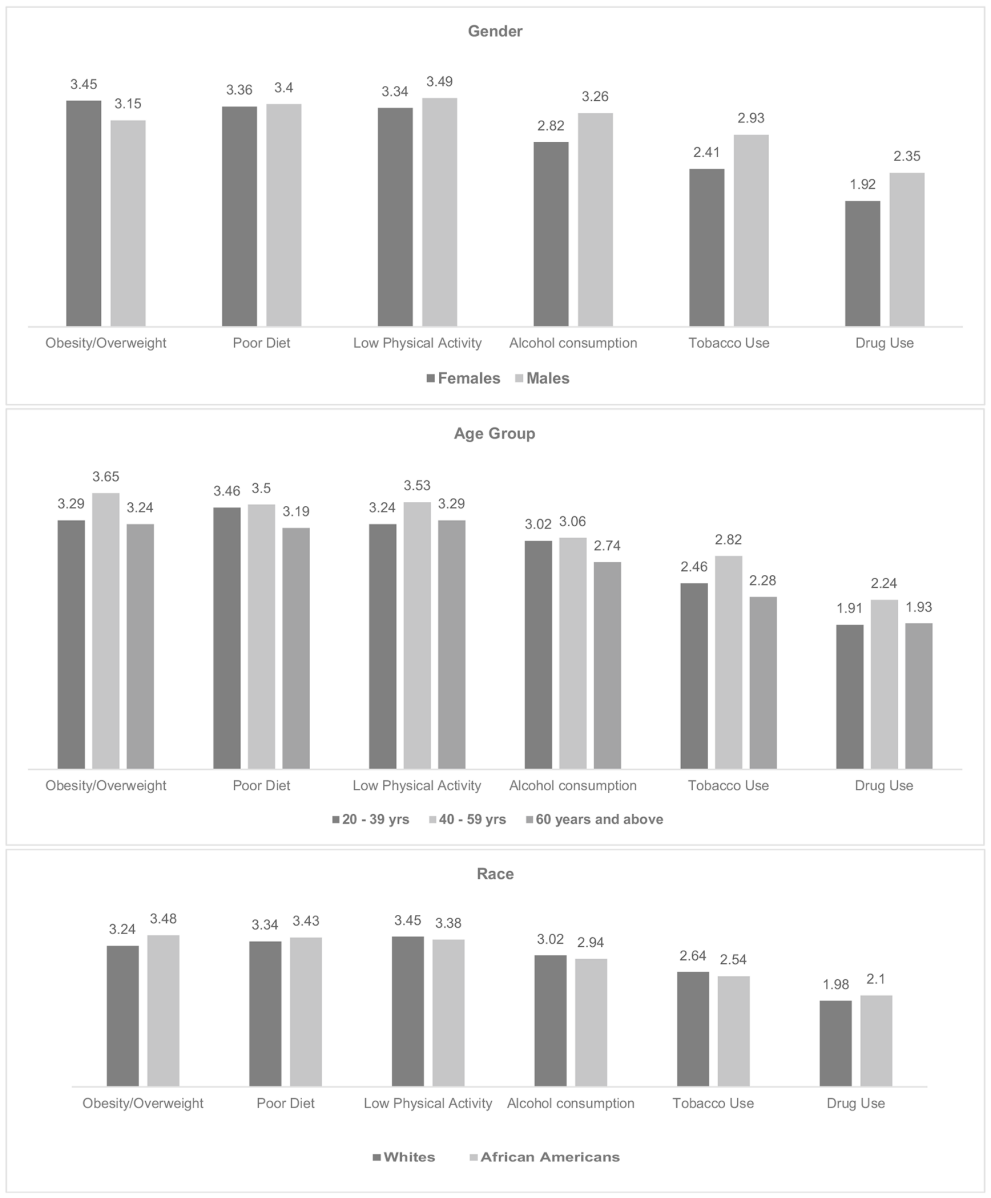
Study participants’ ranking of health problems by demographic characteristics

Overall, the study participants ranked obesity/overweight as their greatest health concern with an average score of 3.38 (on a scale from 1 to 5), followed by poor diet (3.37), low physical activity (3.36), alcohol consumption (2.91), tobacco use (2.53), and the lowest health concern being drug use (2.04). Sub-group analyses revealed that the leading health concern for females was obesity/overweight (3.45); while for males, it was low physical activity (3.49). Alcohol consumption was ranked as a greater health concern among males (3.26) than among females (2.82). Age group differences were also observed, with poor diet (3.46), obesity/overweight (3.65), and low physical activity (3.29) emerging as the most important concerns for adults aged 20–39 years, 40–59 years, and ≥ 60 years, respectively. Similarly, obesity/overweight was also ranked top by more African Americans (3.48) than White participants (3.24) (Fig. 1).

### Participants’ Prioritization of Cancer Education Topics

Overall, priorities for cancer education topics were ranked as follows: “How can one develop cancer” (4.52), followed by “Healthy Eating” (4.49), “How to Access Healthcare” (4.45) “Behaviors that increase cancer risk” (4.44), “Exercise opportunities” (4.42), “Where to go for free/low-cost cancer screening” (4.40), “What happens to personal information when participating in research” (4.23), and the least prioritized topic was “Smoking Cessation” (3.92) (Table 2).

This study also explored attitudes toward cancer and observed that 42.7% of the participants agreed or strongly agreed with the statement, “It seems like everything causes cancer” (Table 2). Subgroup analyses revealed that endorsement of this perception varied by level of education. Specifically, 57.14% of participants with a high school diploma/GED, 44.19% with a vocational/associate’s degree and 41.03% with a bachelor’s/graduate degree agreed or strongly agreed with this statement (*p* = 0.0001).

Sixty-eight percent (68%) of participants disagreed/strongly disagreed with the statement, “There is not much you can do to lower your chances of getting cancer” (Table 2). Subgroup analyses revealed that disagreement with this statement also varied by level of education. Participants with a high school diploma/GED (85.71%), vocational/associate’s degree (65.12%), and bachelor’s/graduate degree (66.67%) disagreed/strongly disagreed with this statement (*p* = 0.0001).

Of the study participants, 51.4% agreed/strongly agreed with the statement that “There are so many different recommendations about preventing cancer, it’s hard to know which one to follow.” (Table 2). Subgroup analyses revealed that participants with a vocational/associate’s degree (58.14%) were most likely to agree/strongly agree with this statement, compared to those with either a high school diploma/GED (50%) or bachelor’s/graduate degree (50%) (*p* < 0.0001).

## Discussion

The SC CADRE initiative aimed to build a bi-directional relationship with the community so that community priorities would guide the center’s research projects and communities would be aware of and engaged in the center’s research. Consequently, this community survey was administered to participants residing in predominantly AA communities across different regions in SC to document perceptions about the health problems (cancer risk factors) prevalent in their communities, their prioritization for cancer education topics, and their attitudes toward cancer prevention. Findings from this study revealed that obesity/overweight, poor diet, and low physical inactivity emerged as the top three major health concerns overall. Thus, community members’ perceptions about their community’s major health concerns are well-aligned with the strong link between these three conditions, as poor diet and physical inactivity, if unchecked, can result in obesity.

In a 2018 study, obesity/overweight was attributed to cause 7.8% of incident cancers and 6.5% of cancer deaths [12]. The proportion of obese adults in the USA increased from 30.5% (between 1999 and 2000) to 41.9% (between 2017 and 2020) [5]. Furthermore, in 2019, an estimated $173 billion was spent on medical costs related to obesity in the USA [5]. Obesity has a higher prevalence among African Americans compared to other racial groups [5, 13, 14]. In our study, African Americans ranked obesity/overweight as their topmost concern, and this finding is supported by a 2022 Centers for Disease Prevention and Control (CDC) report, which revealed that non-Hispanic Blacks in South Carolina had the highest prevalence of obesity (44.5%) in comparison to other racial groups, Whites (32%), Hispanics (35.2%), American Indians (28.6%), and Asians (8.8%) [15]. Furthermore, obesity/overweight was considered more of a problem among women than men in our study, and this aligns with another study that reported a higher prevalence of obesity among women than men [16]. In our study, obesity was a problem among middle-aged adults, and this finding is supported by the CDC report that middle-aged adults had a relatively higher prevalence of obesity (44.3%) compared to young adults (39.8%) and older adults aged ≥ 60 years (41.5%) [5]. Considering that obesity is a major risk factor for cancer and some cardiovascular diseases [14], these reports provide evidence that obesity is a growing public health concern that needs to be addressed urgently.

Poor diet was the second most crucial health problem identified by community members in this study; however, it was a risk factor of most significant concern among young adults compared to other age groups. A 2021 report revealed that fruit consumption among adults in South Carolina was 42.5%, while vegetable consumption (< once daily) was 20.7% (less than half of the adult population) [15]. Socioeconomic disparities such as reduced access to healthy and affordable food options and an abundance of calorie-dense fast food franchises in communities occupied by racial minority groups have been associated with poor food choices and obesity [14, 17]. Young adults are more likely to engage in physical activities either by the nature of their jobs or their exercise routines; however, their busy lifestyles make them more prone to patronizing fast food outlets, increasing their exposure to unhealthy food choices. Similarly, this might explain why poor diet emerged as a more significant health problem among males than females. Therefore, community health education programs and policies must be implemented to increase awareness among African Americans on the importance of healthy eating choices. Policies should also be extended to fast-food franchises to encourage the inclusion of more healthy eating options at reduced costs.

In our study, low physical activity was especially considered a problem among middle-aged adults, males, and White participants. In a 2017 study, physical inactivity accounted for 2.9% of all cancer cases in the USA [12]. Only 47.0% achieved at least 150 min of recommended moderate-intensity or 75 min of vigorous-intensity aerobic physical activity per week (as recommended by the American Cancer Society), and as high as 26% of adults in South Carolina did not engage in any leisure-time physical activity in 2022 [15]. Traffic congestion, inadequate parks, unavailability of pedestrian walkways, leisure or sports facilities, insecurity, and air pollution may have contributed to sedentary lifestyles [18]. This emphasizes the need to enact policies to improve the living environment of racial minority groups by increasing the number of and access to leisure facilities, secured community parks, and pedestrian walkways, among other interventions.

Excess alcohol consumption, tobacco smoking, and substance (drug) use were ranked in fourth, fifth, and sixth places, respectively, overall and across the subgroups. Excessive alcohol consumption (binge drinking) has been associated with an increased risk for several cancers [19], and about 5.6% of cancers were attributed to alcohol consumption in 2017 [12]. Tobacco smoking is the leading preventable risk factor for cancer and has been attributed to causing approximately 19% of incident cancer cases [12, 17]. However, participants in our study ranked tobacco and drug use as the least important health concern. Smoking and drug/substance (opioids, fentanyl) abuse are becoming more popular among young people in multiple communities in the USA. A recent study reported an alarming prevalence of smoking among middle and high school students (11.3% as of 2022) [20], and this calls for sustained efforts to ensure tobacco-control strategies are implemented at community-wide levels to reduce tobacco smoking exposure among adolescents.

Although all the suggested cancer education topics in this study were considered necessary, educating people on the cause of cancer emerged as the topmost priority, revealing that there are still many people who, despite the cancer awareness campaigns in SC, do not have an adequate understanding of the cause of cancer. Also, how to access healthcare, knowing behaviors that increase cancer risk, and where to go for free/low-cost cancer screening were topics that appealed to the participants. The ACS guidelines support risk reduction through several recommendations for nutrition, physical activity, vaccination, and screening [13]. However, studies have reported low compliance rates with these guidelines. A study conducted in South Carolina reported a relatively lower rate of lung cancer screening uptake among Blacks/African Americans compared to Whites [21]. It has also been reported that communities in rural areas may have longer travel distances to screening sites, which could result in lower rates of utilization of screening services [21]. There is also a possibility that some members of minority communities are not aware of screening sites closest to them; hence, it is essential to educate community members on the benefits of compliance with ACS screening guidelines and inform them of where and how to access screening centers. This will facilitate early detection, diagnosis, and initiation of treatment protocols and increase survival rates.

A recent study reported that implementation of a community-based cancer educational program resulted in increased screening knowledge and awareness among their participants [22]. In addition, the study reported a high proportion of community members had intent to talk to a healthcare provider about screening for breast, cervical, and colorectal cancers (85–97%), 88–97% had the intent to get the screening test done in the next 12 months, and 90–97% had the intention to discuss these cancers with their family members and friends [22]. These findings emphasize the importance of targeted culturally appropriate cancer educational interventions and other health programs for racial minority groups to help build their confidence in identifying cancer symptoms and making a choice for healthy lifestyle habits.

Participants in our survey had a relatively high level of education (66% had at least a college degree), but many still expressed some uncertainty about cancer prevention guidelines. For example, 42.7% reported agreement/strong agreement with feeling like everything causes cancer, and 51.4% reported agreement/strong agreement with feeling like there are so many recommendations for preventing cancer; it is hard to know which ones to follow. On a positive note, only13.5% reported agreement/strong agreement with feeling like there is very little you can do to prevent cancer. One reason for these results may be due to the increasing complexity of guidelines for cancer prevention and screening, as guidelines are updated over time to reflect more precise understanding of cancer epidemiology and to incorporate new cancer prevention innovations that become available [13].

### Limitations

A few limitations to this study are acknowledged: first, the sample size was relatively small, particularly for evaluating sub-group differences. Second, other studies have documented the contributions of urbanity and rurality to cancer disparities [17], but we did not assess geographical differences in the prioritization of health problems in our study. Third, the survey did not collect data on study participants’ health behaviors, which could have provided more insight into the perceived importance of health problems, influencing the ranking. Fourth, the findings from this study may not fully represent the population in South Carolina because of the convenience sampling technique employed for participant recruitment.

## Conclusion

In our study, community members identified the health issues and educational priorities that were most important to them. Obesity/overweight, along with the closely related issues of poor diet and low physical activity, emerged as the primary health concerns within our primarily African American study sample. Our study also reporting feelings of uncertainty about cancer prevention guidelines, which may be related to changes to the guidelines over time as new scientific knowledge accumulates. This is an area where further exploration could be valuable. Partnering with communities to identify priority health concerns and critical knowledge gaps and developing intervention programs based on community input can help to increase the success rates of cancer research and public health programs.

## Recommendations

Several recommendations can be provided, based on the results of this study. First, there is a need to develop scalable and streamlined interventions for primary cancer prevention and screening, with a focus on keeping community members abreast of changing guidelines and orienting them to the guidelines that are most relevant to them based on their individual risk factors. Second, programs and resources are needed at the community level to support community members’ strong interest in obesity prevention programming, including support for weight management, nutrition and physical activity. Adoption of a CBPR research approach can help to ensure that input of the community (or target group) is prioritized, from conceptualization to completion of the research and interpretation and translation of results.

## Figures and Tables

**Table 1:** Sociodemographic characteristics of participants (*N* = 179) [1]

Characteristics	(*n*)%
Age (*n* = 172) (mean ± SD)	51.59 ± 16.53 years
< 20 years	1 (0.58)
20–39 years (young adults)	46 (26.74)
40–59 years (middle-aged adults)	56 (32.56)
≥ 60 years (older adults)	69 (40.12)
Gender identity (*n* = 174)	
Male	41 (23.56)
Female	133 (76.44)
Race (*n* = 178)	
African American	129 (72.49)
White	42 (23.59)
Others (Asians, American Indians, and Alaska Natives)	7 (3.92)
Education (*n* = 178)	
Less than high school	3 (1.69)
High school diploma or GED	14 (7.87)
Vocational/associate degree	43 (24.15)
Bachelor’s/graduate degree	118 (66.29)
South Carolina Regions (*n* = 156)	
Low Country	133 (85.26)
Midlands	10 (6.41)
Pee-Dee	12 (7.69)
Upstate	1 (0.64)

**Table 2:** Study participants’ prioritization of cancer education topics and attitudes/perceptions towards cancer prevention

Cancer education topics	Overall		African Americans *N* = 129		Whites *N* = 42	
			
	Average score	Rank	Average score	Rank	Average score	Rank

How can one develop cancer disease	4.52	1st	4.64	1st	4.19	3rd
Healthy Eating	4.49	2nd	4.60	2nd	4.28	1st
How to access healthcare	4.45	3rd	4.56	3rd	4.26	2nd
What behaviors increase cancer risk	4.44	4th	4.57	4th	4.12	6th
Exercise opportunities	4.42	5th	4.54	5th	4.17	4th
Where to go for free/low-cost cancer screening	4.40	6th	4.49	6th	4.17	5th
What happens to personal information when participating in research	4.23	7th	4.48	7th	3.48	8th
Smoking cessation	3.92	8th	3.99	8th	3.83	7th
Study participants perceptions/attitudes towards cancer prevention						
Attitude/perceptions	Strongly disagree *n* (%)	Disagree *n* (%)	Neutral *n* (%)	Agree *n* (%)	Strongly agree *n* (%)	
Seems like everything causes cancer	13 (7.3)	34 (19.1)	55 (30.9)	47 (26.4)	29 (16.3)	
There is not much you can do to lower your chances of getting cancer	36 (20.2)	85 (47.8)	33 (18.5)	16 (9.0)	8 (4.5)	
There are so many different recommendations about preventing cancer, it’s hard to know which one to follow	5 (2.8)	32 (17.9)	50 (27.9)	65 (36.3)	27 (15.1)	

## Data Availability

The dataset generated from the survey are available upon request to the corresponding author.
